# Professional identity formation in public health residents: participation in the vast landscape of practice

**DOI:** 10.1186/s12909-025-08068-9

**Published:** 2025-10-27

**Authors:** Yvonne C. Verlind-Brouwer, Nadieh J.L.M. Taks, Pieter C. Barnhoorn, Sheda Sadrzadeh

**Affiliations:** 1 Research Group ‘Research & Innovation in Public Health Practice based Learning’ (RIPPLE), Netherlands School of Public and Occupational Health (NSPOH), 10th floor, Churchilllaan 11, Utrecht, 3527 GV The Netherlands; 2 Youth Health Care, GGD Hollands Noorden, Alkmaar, The Netherlands; 3 Department of Public Health and Primary Care, Leiden University Medical Centre, Leiden, The Netherlands

**Keywords:** Professional identity formation, Residents, Postgraduate medical education, Community of practice, Scope of practice, Social catalyzer, Legitimate peripheral participation, Personal identity, Public health, Preventive care

## Abstract

**Background:**

With the global shift from curative to preventive care, an increasing number of physicians venture in the field of public health (PH), which could entail changes in their scope of practice and professional identity. Professional Identity Formation (PIF) is progressively regarded as a vital part of competency-based medical education. Although it has been extensively studied in clinical settings, less is known about how physicians’ PIF unfolds outside of traditional clinical environments and what is required to support this process. This study aims to explore how medical residents’ PIF unfolds in the context of PH.

**Methods:**

In this study we used a descriptive qualitative approach, from the perspective of a constructivist paradigm. Cruess’s conceptual framework of PIF in medicine served as a sensitizing framework and supported the interpretation of the data. Eleven PH residents and eight recently graduated PH specialists participated in individual interviews or focus groups. Thematic analysis was performed, along with an iterative process of both inductive and deductive coding.

**Results:**

Participants described the role of a supervisor who is actively involved in the vast landscape of PH and can act as a social catalyzer, as essential in helping them find their professional role. They emphasized the importance of welcoming communities of practice, that give them professional responsibilities to take on tasks aligned with the PH specialty. Participants also expressed the need to reflect on the intersection of personal and professional development. An ambiguous scope of practice and uncertain career perspectives were perceived as inhibiting factors, and could lead to fear of losing legitimacy after residency.

**Conclusion:**

This study offers new insights into the role of the supervisor and the sector-specific challenges in PH residency. To counterbalance the negative impact of an unclear scope of practice on PIF, the authors suggest that more attention must be given to the relationship between personal and professional identity and to the complicated participation in the landscape of practice. While this is particularly relevant for PH residents, it may also apply more broadly to all residents who are confronted with the evolving societal and healthcare demands associated with preventive care.

**Supplementary Information:**

The online version contains supplementary material available at 10.1186/s12909-025-08068-9.

## Background

Public health (PH) is under pressure worldwide. Imminent pandemics, poverty and inequity, an ageing population, mental well-being, and diseases of affluence require a collective approach. Unfortunately, Public Health Medicine (PHM) is often perceived as a less attractive specialty. Despite being a recognized medical specialty, it struggles with an ill-defined identity [1–6]. Its scope of practice – a term referring to knowledge, skills, experience and tasks [7], expected both within and outside the profession [8] - lacks clarity [1, 2, 6, 9, 10]. However, as healthcare systems are shifting priorities from curative to preventive care to address sustainability concerns, there is a growing need for dedicated physicians focusing on PHM and preventive care. This affects not only PH specialists, but also applies to physicians with a main focus on curative care, as they are encouraged to pay more attention to prevention, collaboration and integration of clinical practice with public health initiatives [11]. PH and preventive care are partly intertwined. PH focuses on population-wide health through policies and community-based programs. Preventive care, as a component of PH, encompasses interventions to avert the onset or progression of illness at the individual and community level. This includes primary prevention (e.g., vaccinations, lifestyle interventions), secondary prevention (e.g., early detection and screening), and tertiary prevention (e.g., chronic disease management to prevent complications) [12]. Whereas PH is population-based and institutionally embedded, new forms of preventive care arise within the curative sector, often initiated by individual healthcare professionals. Such initiatives may focus on individuals - for example, through lifestyle counselling or screening - or extend to collective health outcomes, such as community-based projects [13–15].

This fits with a broader trend of healthcare delivery beyond traditional hospital settings [16]. Medical education also reflects this shift, as it increasingly emphasizes community-based settings where students gain experience in health promotion, chronic disease management, and early intervention [17–19]. These educational developments are in line with the role of health advocate, as outlined in the CanMEDS framework for physicians [20].

Shortages in preventive healthcare systems highlight the importance of attracting and retaining physicians in PHM [5, 10]. Retention can be supported by fostering a strong professional identity, as it is associated with enhanced job satisfaction and successful practice by building self-confidence, fostering enjoyment, and strengthening commitment to work [1, 21–24].

Cruess et al. proposed the following definition of professional identity of physicians: “A physician’s identity is a representation of self, achieved in stages over time during which the characteristics, values and norms of the medical profession are internalized, resulting in an individual thinking, acting and feeling like a physician” ([25] p.1447). Professional identity cannot be separated from personal identity [26]. While a strong professional identity contributes to successful professional practice, a mismatch between professional and personal identity can lead to frustration, reduced stress resistance and may even lead to dropping out of the professional role [26–29].

Therefore, several studies have emphasized the importance of incorporating professional identity formation (PIF) in competency-based medical education [21, 24, 30, 31]. Cruess et al. developed a framework of PIF that conceptualizes how medical students and residents form their professional identity [32]. In this framework, PIF is seen as a socialization process, particularly influenced by role models and mentors, clinical and non-clinical experiences, and reflections. Additional factors include the healthcare system, the learning environment, peers, friends, and family. This socialization process is shaped by a community of practice (CoP), which refers to a community of people who share and develop knowledge, beliefs, values, history, and experience [33–37].

In 1991, Lave and Wenger introduced the concept Community of Practice, initially as a learning community within which individuals collaborate to improve their knowledge and skills, and in which learning through participation and social interaction is central [36]. Over the years, the definition of CoP changed and different interpretations arose [38]. CoPs were no longer strictly limited to learning environments but can emerge in various settings such as workplaces and professional organizations. They are characterized by a shared repertoire of procedures, jargon, tools, joint enterprise, and mutual engagement [36–39].

Being welcomed by the CoP strongly fosters PIF, as it provides a sense of belonging and validation [40, 41]. A welcoming CoP facilitates the movement from starting on the periphery and gradually taking on more complex tasks, to full participation within the community. This crucial process is called legitimate peripheral participation [32, 35, 36]. For PH residents, this is particularly important as they work across a fragmented landscape of practice [42], with various stakeholders and CoPs such as municipalities, schools, hospitals and health insurance companies, with associated sector-specific challenges. By successfully integrating PH residents into these diverse CoPs, residents can build the relationships, confidence, and expertise needed to navigate their multifaceted roles effectively, ultimately contributing to impactful professional identity development and PH outcomes.

The shift from curative to preventive care raises questions about how physicians’ PIF unfolds outside of traditional clinical environments and what is required to support this process. However, while PIF has been extensively studied in undergraduate medical education [43] and within curative settings [26, 44–50], its development in PHM remains underexplored, particularly during PH specialty training. Studies on PH identity and physicians specializing in PH do not specifically focus on how professional identity is developed [2–4, 9]. Similarly, in research on developing a professional identity within the PH workforce, physicians are not the group of professionals under study [22, 23].

Public Health Medicine offers a unique lens to explore physicians’ PIF in non-clinical, interdisciplinary, and policy-driven CoPs. These CoPs, often uniting professionals from diverse disciplines (e.g., physicians, epidemiologists, health educators, and policymakers), may serve as empowering environments for socialization and professional identity formation. They might do so by facilitating collaboration across professional boundaries and by sensitizing residents to prevention- and population-oriented thinking, acting and feeling.

Understanding how PIF develops in PHM can illuminate the unique needs of physicians taking on transdisciplinary tasks in non-traditional settings and offer insights into how medical training can adapt to these environments.

This study aims to explore how residents’ PIF unfolds in the context of PHM, examining the factors that influence its development and how it can be supported in non-clinical settings. In doing so, we hope to contribute to a broader understanding of PIF that is applicable not only to PHM but also to other medical specialties and training contexts. Strengthening PIF across diverse settings can ultimately enhance the ability of health systems to attract, retain, and empower a skilled medical workforce.

## Methods

### Context

In the Netherlands, 40–60 residents start PH specialty training every year. In this study, training was offered by the Netherlands School of Public and Occupational Health (NSPOH). The training to become a PH specialist is competency-based and consists of two phases over four years. Weekly, four days of workplace learning, usually with a remote supervisor, are combined with one day of theoretical education. During the first two-year phase, residents specialize in eight different profiles, with Youth Health Care and Infectious Diseases Control forming the largest group. A small minority choose smaller fields, such as Tuberculosis Control, Environmental Health Medicine, or Donor Medicine. After completing the initial phase, the second two-year phase of training focuses on scientific research, consulting and health policy [51]. In this phase, workplace learning is offered by a variety of PH organizations, as well as organizations such as health insurance companies and the military. By the end of the second phase, residents will have specialized as PH specialists. Their responsibilities involve implementing prevention programs, leading health promotion projects, and advising on health policy, often combined with individual healthcare delivery, by which they work at the intersection of both individual and population-based care.

This study focused solely on the second phase of PH specialty training.

### Participants

The study was conducted among PH residents and recently graduated PH specialists. Since the study focused on experiences during training, for which a certain minimum level of completed education is needed, residents whose expected graduation date fell within one year of participation were included. To avoid recall bias, participation was limited to PH specialists who had completed their training no more than a year prior. This led to the inclusion of participants with an (expected) graduation date between 01-12-2022 and 01-06-2025.

### Study design and framework

A descriptive qualitative approach was used, from the perspective of a constructivist paradigm, with semi-structured focus groups and interviews. The constructivist paradigm operates on the premise that knowledge and truth are constructed by and between people and through the meaning they assign to their experiences [52]. We refer to the conceptual framework of Cruess et al., as it connects the PIF of medical students and residents with the process of socialization within communities of practice [32, 53]. The various factors contributing to PIF as mentioned in this framework offered a general sense of direction for understanding PIF in the context of medicine and thus served as sensitizing concepts for the interview guide [54]. The semi-structured approach of the interviews and focus groups provided sufficient room for new input from the participants. We chose focus groups as they allow for an in-depth exploration of participants’ perspectives [55]. Given the personal nature of identity and the fact that not all participants could attend focus groups, the option of individual in-depth interviews was also offered. The combination of focus groups and individual interviews provided the opportunity for collecting the richest possible data from a diverse range of respondents.

### Research team and reflexivity

The research team consisted of YV (PH resident), NT (PH resident until 2024, PH specialist from 2024, trainer at the educational institution) and SS (PhD, PH specialist, epidemiologist, and trainer at the educational institution). The position of the lead author as a colleague resident may have had both a fostering and an inhibiting effect on participation in the study. The researchers were also aware that their position as a PH resident, PH specialist and trainer could influence the interpretation of the data. Efforts were made to set aside personal assumptions as much as possible, but complete bracketing was considered neither realistic nor necessary [56]. Interpersonal reflexivity was achieved by carefully reviewing transcripts manually and discussing interpretations and coding within the research group. The lead author kept a logbook throughout the process of coding and recoding, and included the underlying considerations during meetings and discussions with the other members of the research team. The third author, PB, is a general practitioner (GP), who as a medical researcher has published on the PIF of GP residents [50], and provided the team with a broader perspective on analyzing and transferability of the data.

### Procedure

Participants were purposively sampled from the enrollment records of the educational institute. Based on the (expected) completion date of the training, the relevant cohort was invited via email. The invitation to participate was also posted in a PHM WhatsApp group, and potential participants were approached personally. From the pool of respondents, participants with a diverse range of profile, age, gender, geographical location, and work experience were invited (maximum variation sampling). After registration, participants received an information letter and signed a written informed consent. Date and time were set using an online meeting scheduler. Thereafter, participants received an email with information about the composition of the focus group. Since the composition could affect the perceived sense of safety, the email highlighted the option to withdraw from participation or switch to an individual interview without providing a reason. No participants chose to exercise this option. All focus groups and interviews took place via Microsoft Teams and were conducted and moderated by the lead author, YV. In the focus groups, NT was present as a second researcher to observe the group process, ask additional questions when necessary, and summarize the discussion. The interviews had a mean duration of 44 min, ranging from 28 to 52 min, whereas focus groups lasted on average 80 min, with a range of 74 to 88 min. Given that all focus groups were conducted online, and in order to ensure both audibility of individual contributions and the observation of non-verbal cues, a maximum of six participants per focus group was established. To facilitate sufficient interaction among participants, a minimum of three participants per group was maintained. In all cases, we aimed for a heterogeneous composition of the focus groups. The recording feature of Microsoft Teams was used for audio and video recordings which were transcribed verbatim.

### Data collection and analysis

We started with three individual interviews to refine the initial interview guide. Because an iterative process was employed in which data collection and analysis occurred simultaneously, the interview guide was continuously updated by adding, removing, or rephrasing questions. This allowed the researchers to capture newly emerging gaps and topics that required deeper probing or reframing, ensuring clarity and relevance for subsequent interviews and focus groups. Topics included motivation and professional fit, requirements for becoming a good PH specialist, sources of inspiration, key elements of the educational program, the role of the supervisor, and experiences with workplace learning. The complete set of discussion topics is provided as supplemental material. After the first three interviews, three more interviews were conducted, along with three focus groups.

Data collection took place from November 24, 2023 to July 12, 2024. Data analysis started immediately after the first interview and was repeated after each interview and focus group. Data collection continued until data sufficiency was reached, defined as the point at which sufficient information was available to answer the research question. A total of nine rounds of interviews and focus groups were conducted. Data sufficiency was decided to have been met after the eighth round of data collection. The ninth round was conducted as a confirmatory measure to ensure robustness and thoroughness of the findings and because in this round one of the smaller profiles was represented. This additional round served to verify that no significant new information emerged, thereby strengthening the credibility of the data and confirming that the decision to conclude data collection was well-founded.

A thematic analysis was done, along with both inductive and deductive coding. The initial interview guide was created deductively, using existing literature. Transcripts were initially coded using an inductive approach, as the semi-structured format of the interviews and focus groups allowed participants to introduce new themes. In subsequent transcripts, an iterative approach was utilized, whereby previously identified codes were checked for their recurrence. This process involved a form of deductive verification within the same dataset, aimed at refining and validating the codebook rather than applying codes derived from external theories. Newly identified codes were used to review earlier interviews and focus groups. Codes were grouped and merged into overarching themes. Coding and analysis were performed by two researchers (YV, NT). In follow-up meetings, YV, NT and SS analyzed the themes and reorganized them into a coherent narrative, refining the original framework to provide deeper insights into the research question.

Throughout data collection and analysis, findings were compared with the literature. In this process, where codes and themes were not only “seen” but also “seen as,” inductive and deductive coding were integrated [57]. The coding program MAXQDA© 2022 (MAXQDA qualitative data analysis software) was used for data analysis. We applied the Consolidated Criteria for reporting qualitative research (COREQ) [58].

### Ethical approval

Approval for this study has been obtained from the Ethical Review Board of the Netherlands Association for Medical Education (NVMO-ERB), protocol number 2023.4.2.

## Results

**Fig. 1:**
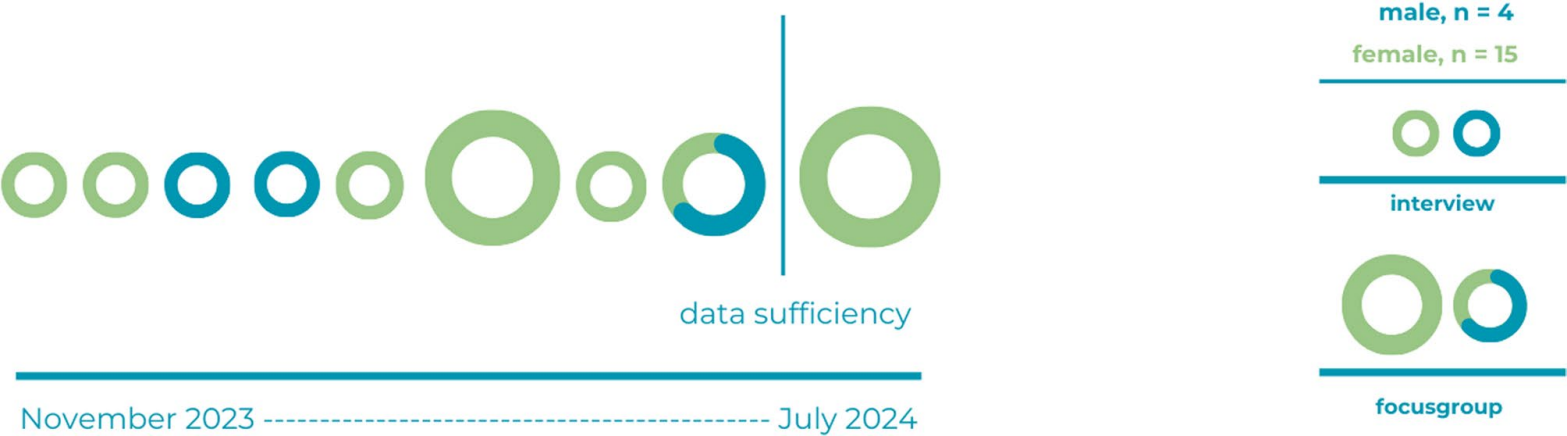
Timeline with interviews and focus groups

Three focus groups were conducted, each consisting of three to five participants, together with six individual interviews, resulting in a total of nineteen participants. Figure1 shows the timeline with an overview of the interviews and focus groups.

Eleven PH residents and eight PH specialists, spread across thirteen organizations, participated in this study. Ages ranged between 31 and 49 years. In seventeen participants, Youth Health Care or Infectious Diseases Control were the underlying profiles in which residents specialized during their first phase. Two residents had a background in other profiles. Table1 presents the characteristics of the participants. To prevent traceability to individual respondents, distributions of participant characteristics are not detailed further.

**Table 1:** Participant characteristics

Characteristic	Category/Value	*n*
Training phase	PH resident^a^	11
	PH specialist^b^	8
Profile	Youth Health Care	12
	Infectious Diseases Control	4
	Combined Profile	1
	Tuberculosis Control	1
	Health Policy Advisor	1
Gender	Male	4
	Female	15
Age	Median (range)	39 (31-49)

^a^expected graduation date within one year of participation

^b^graduation date no more than one year prior to participation

Our participants identified several aspects of PH specialty training that could either foster or inhibit PIF. These could be categorized into four major themes, all of which were related to participation in the landscape of various CoPs:

- ambiguous scope of practice
- legitimacy and role assertion in the workplace
- supervisor as a role model, coach, and social catalyzer
- personal identity and tailored training

Using expressions and quotes from the participants in order to stay close to their words, we will explore each theme. Here, interviews and focus groups will be indicated by I and FG, respectively, followed by the round number n.

### Ambiguous scope of practice

For PH residents, becoming a PH specialist signified a desire to engage in more overarching work, assume greater policy responsibilities, collaborate with external stakeholders, or, alternatively, concentrate more on education or scientific research. They mentioned, however, that their employers and organizations did not perceive a clear or well-defined role for PH specialists in these areas.

*“I have observed that within our organization we are still very much exploring the specific role and position of the PH specialist, and that, at the individual level, there is also an ongoing search for how to shape this role and how to be a good PH specialist”. (FG1)*

Participants worried about the ill-defined identity of PHM and perceived their role recognition as unclear, not only in others but also in themselves. They struggled to define their scope of practice: the specific tasks and job responsibilities of the PH specialist.

*“Even now, when someone asks me “what is a PH specialist?", I still don't quite know how to answer that.” (FG1)*

The unclear distinction from other professionals in PH complicates the positioning and the recognition that comes with it.

*“But what our directors also say is that they don’t really know what a PH specialist actually does. What difference can you make? What sets you apart from a health promotor, what makes you different from a Youth Health profile physician? And how can I actually utilize you?” (FG1)*

This prompted questions such as: What do I truly wish to achieve as a PH specialist, and how do I intend to give shape to it? What capabilities does this specialty entail, and what optionsare available?

*“But what makes it very difficult, in my opinion, is that in our organization there is no policy on what you are supposed to do after PHM registration. So you don’t really know either what to grow into. So I feel like I'll just be done soon and then nothing will change.” (I2)*

This ambiguous scope of practice affected participants’ confidence in their future opportunities. Although there were individual differences, many of them felt *“pessimism”(FG1)*, *“discouragement and hopelessness”(FG2)* about their career prospects. Due to the additional limited job openings, finding their place was *“a struggle”* for many *(FG1, FG2, FG3*).

Following the completion of their training, some residents were able to continue working within the organization where they had undertaken their workplace learning. Opportunities to assume new tasks aligned with the PH specialty after graduation were highly dependent upon the agreements established with employers regarding the scope of permissible work, and *“many challenges”(I6) *remained in this respect.

Others, in contrast, were required – or chose themselves - to seek new employment, which could be challenging.

*"I also feel there are still few vacancies for PH specialists - real positions dedicated to this role." (FG2)*

### Legitimacy and role assertion in the workplace

Within the group of participants, starting positions varied. Most residents started at the periphery of the CoP, whereas some others had already gained several years of work experience with almost full participation and legitimacy in various CoPs, which made them *“already feel like a PH specialist before residency” (FG1). *Yet, for most residents, being in training offered a new opportunity to join meetings and projects that would otherwise have been challenging to attend or would have been entirely inaccessible. It gave them *“legitimacy to take things on”(FG2),* to connect with others and to ask if they could join in.

At the same time, there was a lot of fear “*that when you are finished, you will have lost that legitimacy that residency gives you” (FG1). *Participants felt they must “*strategically position themselves in the organization” (FG1)* to secure their position after completing residency.

*"In our organization, once you have completed the training, it is your own responsibility to ensure that you are able to take on tasks that align with the PH specialty […]. This means that, after completing the program, you do not automatically have the legitimacy to be a PH specialist - at least not with us." (FG1)*

The organization that offered workplace learning served as the primary setting for practical training, making it the most critical CoP for residents. Participants felt they had to connect the theoretical education regarding strategy, policy, innovation, and governance with workplace learning, and therefore needed *“practical things to apply them to […], joining a particular project or taking the lead on something”(I4)*. They indicated that gaining “*responsibility” (I1, I3)* and*“confidence”(I5, FG2, FG3) *from their employer was crucial for their PIF.

Some organizations gave residents opportunities to develop themselves in various areas, which contributed to *“also feeling more and more like a PH specialist” (I6). *Really “*adding something, getting things done” (I4) *in PH reinforced this feeling.

In other organizations, residents had to figure it all out for themselves, and when there was overlap in tasks with other PH professionals, taking on a specific position by a resident could be perceived as “*competition, rather than as an addition”(FG2). *Specifically in policy tasks, not all organizations were convinced that they “*belong to the PH resident” (FG3), *making role assertion and participation in this area very difficult.

*“There are people who do not receive any opportunities at all from their organization, no matter how hard they fight [.......]. Actually, it's quite an individual struggle and I think that's a pity. It shouldn't be that way.” (FG1)*

PH residents deal, not only with their own organization, but also, for example, with municipalities, schools, hospitals, and health insurance companies, which form a vast landscape of practice. This meant constantly engaging with and becoming part of new communities of practice, requiring effort and perseverance *“to get a seat at the right table” (I5). *Speaking the language of managers and executives as well as that of local governments and health insurers, enabled them “*to participate in discussions and contribute ideas” (FG3)* and see *“that your medical perspective is just one argument in the whole pie of arguments”* (*I1)*.

However, the extent to which residents felt welcome in these communities varied greatly. In some CoPs, residents could easily position themselves, participate and cooperate, and assert their professional role, while in others they could *“hardly get involved”(FG2) *and stakeholders made them feel *“actually being constantly dismissed” (FG1) *and *“immediately seen as meddlers” (FG1)*.

### Supervisor as a role model, coach, and social catalyzer

Within workplace learning, specific educational tasks are reserved for the supervisor, who can fulfill various roles. PH supervisors served as role models solely when they were actively involved in clearly defined collective preventive care tasks, as opposed to individual care. In doing so, supervisors provided residents with opportunities to observe and internalize professional behavior, norms, and values within interdisciplinary settings and outside their own organization.

“*I have a supervisor who inspires me greatly [...] and she is currently very much in an advisory role with the municipal health service, also doing a lot of national and overarching work, which inspires me immensely.” (FG3)*

According to the participants, supervisors often took on the role of a coach; someone you can consult, and with whom you evaluate and reflect.

Participants mentioned their supervisors were important in supporting them by expressing confidence, encouraging them to explore new possibilities and to broaden their perspective, advising on future plans, and reflecting with them on the goals set or achieved. Through feedback and guided coaching, supervisors supported residents’ reflective processes and fostered self-awareness. Supervisors also offered recognition and validation of residents’ roles and competencies.

* “And what also helped me is that at a certain point my supervisor just started saying: well, as far as I'm concerned, you're already a PH specialist. You can always get a little better and tighten up a bit, but as far as I’m concerned, you've actually already made it. […]. And that also helps, of course, that someone just literally says that to you every now and then.” (FG1)*

But most importantly, it was necessary for the supervisor to be a social catalyzer, “*making their network available to you”(I2, FG1). *Acting as a social catalyzer meant that supervisors facilitated collaboration by bringing residents and stakeholders together and helping residents find their professional role in the vast landscape of PH. As not all supervisors participated in the vast landscape of practice and also *“struggled with the identity of the PH specialist themselves” (FG2)*, participants mentioned the possibility of assigning the most appropriate supervisor to a particular resident, based on individual needs.

*“Within our organization, you could really see a difference emerging between PH residents who had a supervisor involved in policymaking […] and had clearly defined PH specialist tasks. It was evident that those residents just got more chances.” (FG1)*

### Personal identity and tailored training

Participants frequently articulated a connection between their professional identity and their personal identity. Several aspects were mentioned within this context, including the autonomy to shape one's work in alignment with personal inclinations, to adopt a medical leadership style consistent with one’s personal identity, to think beyond predefined frameworks, and seeing things from a broad perspective.

*“I’m quite broadly oriented by nature and I always like to take a step back and consider the bigger picture. So, I think that’s just part of who I am.” (FG3)*

For some participants, it was clear how the PH specialty fits them personally.

*“ I suddenly felt: Ah, okay, everything comes together now and who I am, and what kind of doctor I want to be, and what I want to achieve, and what my goals are within my professional career, I can put all in this [….] So actually who I am, I can completely express in .... in being a PH specialist.” (I3)*

Residents needed freedom of choice regarding PHM topics they engage with. This choice could involve selecting their own area of expertise, deciding whether to take on cross-functional roles, whether or not to undertake management tasks, or whether to focus on deepening or broadening their skills.

*“Yeah, and within the training program, you have the opportunity to organize your hours and assignments around things you find interesting to do. I think that’s really important too – that not all the assignments are completely fixed, or that you’re told: you have to spend this many hours on policy here, or that many hours there. Instead, you can sit down with your supervisor and figure out: what suits you, and what do you enjoy doing within the setting where you are doing your training” (I3)*

The direction in which participants tailored their training were related to (i) character traits such as *“(not) being a great manager” (I4), or “being a true connector” (FG3), * (ii) a strong inner drive, sometimes rooted in and shaped by childhood experiences, for instance *“achieving health gains by influencing the living environment” (I3, I5),*or (iii) previous career experiences, like *“missing PH issues in clinical care” (FG2)*. But for several residents, it was still a journey of exploration to “*figure out what suits me best” (I6).*

*“You are working on those competencies and so on, but who you are and how you are formed during the training ... not so much.” (I2)*

During training, there was ample room for reflection on the theoretical education and the learning process, but it would have been valuable to allow more consideration of *”how your personal life, your personal development and that professional development, how they go together”(FG3).* At the end of the training, it all comes down to the question *“but who am I now? I'm done now and who am I now?” (FG2).*

## Discussion

With this study, we aimed to gain insight into the PIF of PH residents, to empower future physicians who are needed to meet the changing demands of society and healthcare regarding preventive medicine. Our results indicate that PH residents’ PIF is primarily determined by their participation within the various CoPs. Participation acted as a common thread throughout the themes. This was reflected in the ability to connect theoretical education to practical assignments, gaining experience within the workplace, feeling welcomed, and being involved in new CoPs. Limiting factors were the poor-defined scope of practice and the associated uncertain career prospects. Below, we will discuss our findings and how they relate to the literature. In the final part of the discussion, we reflect on the interrelationships among the major themes of this study.

### Ambiguous scope of practice

An ambiguous scope of practice seems to hinder PH residents in establishing themselves as members of the CoP . While they are still in the process of identifying their added value themselves – making it difficult to confidently assert their professional role - they are additionally confronted with unfamiliarity or even resistance concerning their involvement. An ambiguous scope of practice also affects their confidence regarding career opportunities. A paradox emerges here: despite the growing societal and healthcare demand for a stronger focus on disease prevention and the sustainable management of healthcare expenditures, PH specialists – for whom preventive care is a core responsibility – encounter difficulties in securing appropriate responsibilities and tasks. This often necessitates proactive job crafting on their part.

The relationship between an unclear scope of practice, a poor professional identity, the blurred distinction of PH specialty in relation to other specialties, few job openings, and the vicious cycle that arises between these factors, has been described by several researchers [1, 9, 10, 22, 23, 27]. Zweigenthal et al. point to the need for professionals within PHM to carve their own career paths as a result of PHM’s poor profile [1] , and relate the unclear scope of practice and the overlap in competencies with other PH professionals as a threat to future PH specialists’ career paths [10] . Research on the identity and scope of practice of the PHM specialty has also been conducted by Jadotte et al. [2, 3, 59]. In his article ‘The foci of the Public Health Preventive Medicine Specialty’, he mentions the “lacking focus by the public, given the breadth of the specialty” ([3]p.2). Our research confirms the difficulties concerning career prospects resulting from the ill-defined identity of the PHM specialty, as described in these articles.

The strong emphasis our study places on the scope of practice and job openings is not commonly reflected in the literature on PIF in clinical or GP residents [26, 44–50, 60], and thereby highlights a specific aspect of PH residency.

### Legitimacy and role assertion in the workplace

Lave and Wenger [36] describe that access to the CoP is crucial but also problematic. Fully becoming a member of a CoP requires access to a wide range of activities and other members of the community, and to information, resources, and opportunities for participation. Particularly in the latter, there seem to be constraints in PH residency due to unclear or even negative perceptions about the role of the PH specialist among the workforce, management, and other professionals [1, 9, 27].

This reluctance makes it difficult to apply theoretical knowledge in practice, which frustrates residents’ socialization process. This causes a lack of a sense of belonging to the community and carries the risk that PH residents remain stuck on the periphery of their CoP. At the same time, the literature shows how beneficial it is for PIF when organizations offer generous opportunities for participation, and when residents are given professional responsibility and confidence [27, 35], as is underlined in our study.

In the vast landscape of PH, with its various stakeholders, participation can be even more difficult. Given that some stakeholders explicitly question the relevance of the PH specialist, it is important to focus on what is discussed regarding interprofessional education: residents should consider how their practice might be relevant for other professionals, how activities can be coordinated, and how collaboration can be structured [4, 42, 61].

Upon completing their training, many PH residents fear losing the legitimacy to fulfill the responsibilities associated with the professional role of the PH specialist. This fear is remarkable and mainly due to organizations and CoPs not knowing how to deploy PH residents after they are graduated PH specialists. This presents not only a practical challenge for PHM but also a challenge in terms of training PH residents.

Comparing these findings with the main factors in the framework of Cruess et al. [32], our research places greater emphasis on the influence of the learning environment, healthcare system, and attitude of others on the socialization process.

Despite all the ambiguity and uncertainty about their scope of practice, there are residents who brought about improvements in their organization and in collaboration with stakeholders. This illustrates that “individuals are not only shaped by the CoPs that they participate within, but themselves change the CoP” ([35] p.7).

### Supervisor as a role model, coach, and social catalyzer

Among others, Cruess et al. [32] and Snell [40] emphasize the importance of role models [27, 46, 50]. In GP residency, it is often the supervisor who serves as a role model [50, 60]. But while there is a clear master-apprentice relationship in clinical and general practice, with role models and mentors playing a central role in the transfer of knowledge [37, 46], this relationship is nearly absent among PH residents, who usually have a remote supervisor. Regardless, PH residents described having clear role models, with supervisors being a possible role model only if they are identifiable in collective preventive care tasks and do not remain focused solely on individual care.

The perception of the supervisor as a mentor or coach aligns with the literature [32, 50], and is not surprising, as supervisors observe and reflect on residents’ skills and provide guidance for their practical assignments.

This study highlights the third role of a supervisor, being a social catalyzer, as the most important. To apply the knowledge gained and take on collective preventive tasks, PH residents need someone who is familiar with the CoPs and provides them with access to their network. Residents with a supervisor who fulfilled this role, clearly had more opportunities. To the best of our knowledge, this role has not been previously described.

In being a role model or coach, supervisors influence PIF more or less independent from other organizational actors. However, their role as a social catalyzer may entail greater complexity as there is both overlap with and reliance on often long-established collaborative relationships within the organization and with external stakeholders.

### Personal identity and tailored training

Veen and de la Croix [62] point out that PIF as a socialization process within the CoP-model entails the risk that PIF is no longer about identity, but about conformity. In our study, we do not identify a tendency toward conformism, but residents valued the ability to tailor their education and responsibilities, which corresponds to the need for authenticity [49].

Some residents previously developed a strong inner drive to make an impact within PH before PH specialty training started and had well-defined, crystallized ideas about how they wanted to incorporate their ideals and expectations into their profession. We recognize in this Korthagen’s model of layers of personality [63], which Barnhoorn et al. adapted into a framework for the medical context [64]. In this model, a person's ideals (mission) form the innermost layer of an onion, surrounded by layers of identity, beliefs, competencies, behavior, and environment. Having a powerful inner motivation appeared to be highly beneficial in overcoming obstacles to participation.

Residents appreciated reflections on their learning process, but they mainly emphasized the need to reflect on themselves as a person and how they are formed during residency. Several residents indicated this should be more thoroughly attended to during training, aligning with Cruess’s recommendation that *“identity formation must be addressed explicitly in the formal curriculum”* ([31] p.1). It is also in accordance with Rees and Monrouxe: *“medical students, trainees and trained doctors should regularly engage in conversations about and for their identity development.”* ([21] p.203).

### Intersection of educational structure, scope of practice, workplace dynamics, supervisory support and personal identity

The themes of this study are interrelated. Workplace dynamics shift when residents enter the setting and in the absence of a clearly articulated scope of practice, managers as well as colleagues from related disciplines may be unsure how to engage with the PH resident.

While guidance and endorsement from the supervisor can promote collaboration and facilitate integration into the CoP, lack of supervisory support may exacerbate uncertainty and impede participation. The fact that some supervisors themselves are impeded by the ambiguous scope of practice of the profession and have not themselves established collaborative relationships outside their own profession or organization, leaves this process exposed in certain training contexts. Similarly, the personal identity and the prevailing nature of the CoP may interact with and influence one another. Some residents articulated a clear and robust vision regarding the role of the PH specialist, thereby operationalising the often ambiguous scope of practice, and, in doing so, are able to mobilise the support and engagement of other professionals.

PH residents shape and reshape their professional “selves” through ongoing interactions within the educational institution and communities of practice. Through theoretical knowledge, reflection, role assertion, and participation within the vast landscape of practice, they navigate their professional roles and self-conceptions. Our study illustrates PIF in PHM as a multi-dimensional process of socialization, that is highly dependent on the legitimacy to participate and the ability to acquire a position within the various CoPs, with the supervisor acting as a social catalyzer.

The competency-based educational program, in which practical professional experience (four days of workplace learning per week) and the integration of theoretical instruction with practical assignments play a central role, could support the PIF of PH residents. This may be achieved by allocating space in the curriculum for reflection on residents’ personal and professional identity, preparing supervisors to recognize the importance of their role as social catalyzers via teach-the-teacher programs, during accreditation visits evaluating the extent to which organizations offer a welcoming environment for residents, and allowing students the freedom to engage with topics that most effectively foster the development of their professional identity.

A learning environment that supports professional identity formation in all its complexity emerges when an educational institute encourages residents to integrate theoretical knowledge with professional practice, grants them the autonomy to select topics closely aligned with their interests and personal identities, is complemented by CoPs that welcome residents to navigate their professional roles and apply theoretical knowledge within the workplace context, and is guided by supervisors who, beyond serving as coaches or role models, act as social catalyzers and encourage residents to engage in reflection not only on their knowledge and skills, but also on the kind of physician they aim to become within the vast landscape of PH.

### Implications

To counterbalance the negative impact an unclear scope of practice has on PIF, the authors suggest that more attention should be given to the complicated participation in the landscape of practice during PH residency. This calls for extra vigilance among supervisors to act as social catalyzers when necessary and foster participation wherever possible. The challenges of participation apply to PH residents, but are similarly likely to affect other residents who cross boundaries between clinical and preventive care and want to fulfill their role as health advocates in communities and society, as outlined in the CanMEDS framework.

This implies, just as for PH residents, that new domains must be explored where their roles are not always well-defined, new ways of working need to be adopted, and supervisors are needed to guide them through this process. Therefore, we expect that our findings concerning participation in new non-clinical CoPs are not only relevant for PH residents, but are transferable to other residency settings. The need to reflect on PIF may be particularly important for all residents exploring their path in PH and preventive medicine, as they could be pioneers in the current changing healthcare landscape.

Further research on factors influencing socialization and participation outside of traditional clinical environments can enhance our understanding of PIF in these settings. In addition, we recommend further investigation on the role of social catalyzers and on the development of PH specialists’ professional identity in the years after graduation, especially regarding their fear of losing legitimacy.

Subsequent exploration might investigate the ways in which uncertainty tolerance affects PH specialists’ self-efficacy, resilience and decision-making and how it shapes their participation and positioning within communities of practice.

### Limitations

To collect the richest possible data from a diverse range of respondents, we invited residents and recently graduated specialists from all profiles. Our participants belonged to only four of the eight possible profiles, with Youth Health Care and Infectious Diseases Control being most strongly represented. This representation is in line with the distribution across the study population, but does not eliminate the possibility of skewing the results. This risk is amplified by the fact that four small profiles were not represented at all. This study did not specifically investigate the various profiles from the first phase, but our results do not indicate differences between participants from these profiles regarding their professional identity formation. To more definitively assess the influence of training and work experiences prior to the second phase, in which they are becoming PH specialists, further research focusing explicitly on this issue with a larger number of participants from each profile would be required.

Participation may be biased by individuals with an affinity for the topic. This might have skewed the results in both ways as residents with a strong PIF as well as a weak PIF might not see the relevance of the topic and thus did not sign up for interview or focus group participation.

Belonging to a minority group can influence PIF. Although minorities were represented in our study, we did not differentiate between them in this regard.

To study the PIF of PH residents, we assumed their residency shows similarities to other specialties who are moving toward the development of preventive care. We did not conduct prior research on this assumption, so transferability may be limited.

## Conclusion

Health systems and societies are at the beginning of what is becoming a significant shift toward prevention rather than cure, in which more and more physicians will need to take on new roles. This study aimed to advance understanding of how these developments impact the PIF of residents who are shaping their career during this transition. In the context of PH residency, PIF seems to be significantly influenced by the opportunities to socialize and participate in the vast landscape of various CoPs. These opportunities are primarily fostered by the workplace, the supervisor, and residents’ personal identity. Contrarily, an ambiguous scope of practice and associated uncertainty about career perspectives were perceived as inhibiting factors. We expect that the challenges of participation apply not only to PH residents, but are likely to affect other residents in new, not exclusively clinical, CoPs as well.

## Supplementary Information

Supplementary Material 1. Interview guide

Supplementary Material 2. COREQ standard

## Data Availability

The datasets used and/or analyzed during the study are available from the corresponding author on reasonable request.

## Abbreviations

CoP: Community of Practice
FG: Focus group
GP: General Practitioner
I: Interview
NSPOH: Netherlands School of Public and Occupational Health
PIF: Professional Identity Formation
PH: Public Health
PHM: Public Health Medicine
