# Supplementary material for: Professional identity formation in public health residents: participation in the vast landscape of practice
Source: BMC Med Educ. 2025 Oct 27;25:1503. doi: 10.1186/s12909-025-08068-9 (PMC12560368; doi:10.1186/s12909-025-08068-9)
Supplement: Supplementary file 1 — Supplementary Material 1. Interview guide [file 12909_2025_8068_MOESM1_ESM.docx]

INTERVIEW GUIDE

**Title**

Professional Identity Formation in Public Health Residents: Participation in the Vast Landscape of Practice

**Research question**

How does the development of PH residents’ professional identity unfold?

1. What made you feel the profession of Public Health (PH) specialist suits you?
2. What did you - or do you still - need to become a good PH specialist?
3. Who (or what) inspired you to pursue this profession?
4. Which aspects of the educational program do you consider the most important in supporting your professional development from physician to PH specialist?
5. How do you perceive the role of your supervisor in this development?
6. How do you experience workplace learning in your own or in different organizations?
7. When was the first moment you truly felt like a PH specialist?

- Have there been moments or periods when you felt like you did not belong to this professional field?

8**.** Are there any topics overlooked?
